# Open educational resources

**Published:** 2015

**Authors:** Daksha Patel, Sally Parsley

**Affiliations:** E-learning Director: International Centre for Eye Health, London School of Hygiene and Tropical Medicine, London, UK.; Learning Technologist for OER: International Centre for Eye Health, London School of Hygiene and Tropical Medicine, London, UK.

**Figure:**
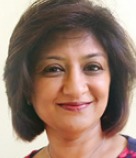
Daksha Patel

**Figure:**
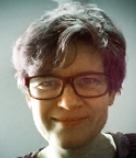
Sally Parsley

## What is open education?

Historically, ‘open education’ has involved making education more accessible, whether by lowering cost or by enabling delivery at a distance.^1^

In our technological age, open education has become a global sharing of knowledge and ideas through the Internet.

## What are open educational resources (OERs)?

OERs are learning materials such as textbooks, presentations and quizzes shared under an **open copyright license,** such as Creative Commons, or placed into the **public domain.** This means that both educators and users (learners/students) can have access for free, and educators can directly reuse, adapt and republish content without having to seek permission from the original author.

## The benefits of open education and OERs

- **Promotion and re-use of existing high quality content and practice.** This means not having to re-invent the wheel.
- **Breaking down barriers** (age, culture and cost). OERs on the internet reach greater number of people which increases impact.^2^
  **Figure:**
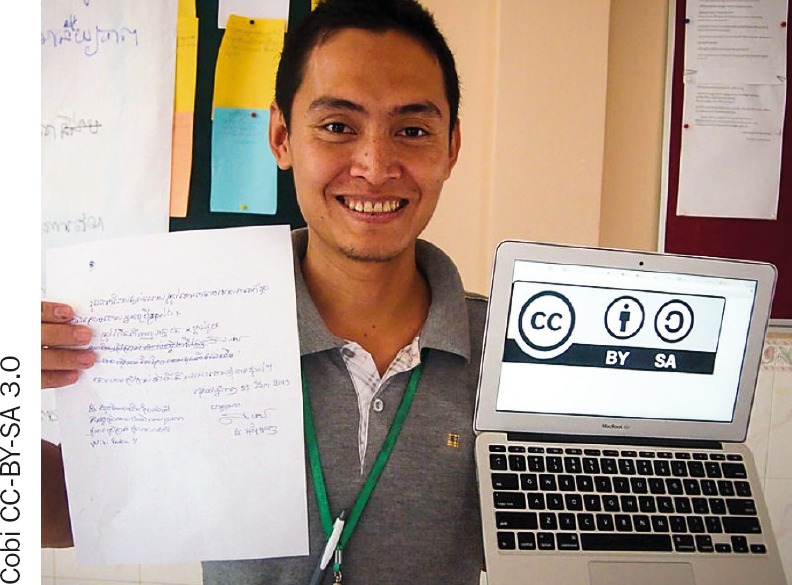
Creative Commons licenses are used around the world, such as pictured here in Cambodia.
- **Continuous quality assurance.** By sharing materials as OER, educators can work collaboratively to improve their material and set quality bench marks.^3^
- **Career development.** By inviting comment and collaboration, educators gain access to new ideas for practice and career enhancement.^1^
- **Equalising access.** OERs support educational facilities with limited faculty and infrastructure.

## What is ICEH doing in open education?

Using an open education approach and with support from Seeing is Believing, the International Centre for Eye Health (ICEH) adapted content from its existing training programmes and from the *Community Eye Health Journal* to create an OER called ‘Global Blindness: Planning and Managing Eye Care Services’. The purpose of this OER is to equip and support eye health providers to plan and implement local strategies to eliminate avoidable blindness and visual impairment, particularly from cataract and refractive error.

The OER was pilot tested with success in Kenya, Ghana and Botswana, and then launched on Future Learn as an Open University partnership. It ran as a six-week interactive course with over 3,500 participants in more than 80 countries and comprised videos, articles and quizzes.

## Using the Global Blindness OER in your setting

To get access to the OER content, email us on eyeplan@lshtm.ac.uk.

- Do the course as a team project in your hospital/clinic to improve services.
- Introduce and adapt the OER into your medical and post-graduate curriculum.
- Provide us with feedback to improve the content.

The fact that the course is an OER means that you do not need to worry about plagiarism or copyright issues and learning can be shared. ICEH has plans to develop and deliver further OERs funded by the Queen Elizabeth Diamond Jubilee Trust.
